# Eradication of tetanus

**DOI:** 10.1093/bmb/ldv044

**Published:** 2015-12-07

**Authors:** C. L. Thwaites, H. T. Loan

**Affiliations:** † Oxford University Clinical Research Unit, Hospital for Tropical Diseases, 764 Vo Van Kiet, Ho Chi Minh City, Vietnam; ‡ Nuffield Department of Medicine, Centre for Tropical Medicine and Global Health, University of Oxford, Oxford, UK; § Hospital for Tropical Diseases, 764 Vo Van Kiet, Ho Chi Minh City, Vietnam

**Keywords:** tetanus, elimination, maternal, neonatal tetanus

## Abstract

**Introduction:**

The causative agent of tetanus, *Clostridium tetani* is widespread in the environment throughout the world and cannot be eradicated. To reduce the number of cases of tetanus efforts are focussed on prevention using vaccination and post-exposure wound care.

**Sources of data:**

Medline, Pubmed and Cochrane databases; World Health Organization and United Nations Children's Fund publications.

**Areas of agreement:**

The maternal and neonatal tetanus elimination initiative has resulted in significant reductions in mortality from neonatal tetanus throughout the world.

**Areas of controversy:**

Although there are few data available it is likely that large numbers of children and adults, particularly men, remain unprotected due to lack of booster immunization.

**Areas timely for developing research:**

It remains unclear how HIV and malaria affect both responses to vaccination and transplacental transfer of antibodies or how this might affect timing of vaccination doses.

## Introduction

Tetanus is caused by a potent neurotoxin produced by the bacterium *Clostridium tetani*. The word ‘tetanus’ is derived from the Greek ‘tetanos’ meaning to contract. The clinical features of tetanus have long been known: there are descriptions in the Edwin Smith Papyrus of ancient Egypt (1600 BC), the writings of Hippocrates in ancient Greece (400 BC) and in the Ayurveda texts of ancient India (400 AD).

More recently the precise mechanism of action of tetanus toxin has been characterized. *C tetani* spores are present in the soil and wider environment throughout the world. Once in a suitable anaerobic environment such as a contaminated wound, the spores are able to germinate and the bacteria multiply, releasing tetanus toxin. In approximately 20% of cases there is no obvious entry site and often minor cuts and abrasions form the portal of entry.^1^ In the case of neonatal tetanus, the entry site is the umbilical stump and traditional birth practices such as cutting the cord with grass or applying cow dung increase the likelihood of infection.^2^

The toxin is composed of heavy and light chains, linked by a disulphide bond. The heavy chain mediates toxin uptake and transportation whereas the light chain is responsible for its pathological activity. The toxin is taken up from motor endplates and undergoes retrograde transport into the central nervous system.^3–5^ The precise mechanism by which it crosses the synapse is still unclear but the N-terminal of the heavy chain mediates light chain entry into the pre-synaptic inhibitory neurone and where it cleaves synaptobrevin (vesicle associated monophosphate 2).^6^ This molecule is necessary for pre-synaptic docking and subsequent neurotransmitter release of synaptic vesicles thus the tetanus toxin disinhibits the motor neurones. This results in unrestrained alpha motor neurone discharge and muscle contraction. Similar actions are assumed to occur in the autonomic nervous system.

As tetanus spores are present throughout the world, and are resistant to heat and chemicals,^7^ unvaccinated individuals sustaining wounds contaminated with *C tetani* spores are always at risk of the disease and herd immunity plays no part in tetanus prevention. Furthermore due to their continued presence in the environment, complete eradication is unlikely and cases will continue to occur.

Effective immunization programmes and good post-exposure prophylaxis means tetanus is rare in developed countries, although cases continue to be seen.^8,9^ In the UK only seven cases occurred in 2013,^10^ but in other parts of the world the disease is more common. Less than 10% of cases of tetanus are reported and the global incidence of tetanus is unknown. Even in the USA it was estimated that less than half of cases were reported to the Centers for Disease Control.^11^ Sizeable case-series continue to be reported in the international literature and figures from 2013 show an increase in the total cases of tetanus recorded by the World Health Organization.^12,13^

More accurate data are available for neonatal tetanus, the elimination of which has been the focus of a major initiative by the World Health Organization and its partners. This initiative, launched at the World Health Assembly in 1989 aimed to eliminate neonatal tetanus by 1995. At that point an estimated 800 000 neonates a year were affected by the disease with a mortality rate of 6.7 per 1000 live births.^14^

## Areas of agreement

Mortality from tetanus varies between approximately 10 and 80%,^13^ but the disease is completely preventable by vaccination and post-exposure prophylaxis. Tetanus toxoid vaccination became available in the UK in the 1950s and routine vaccination began in 1961. A combined ‘DTP’ diphtheria-tetanus-pertussis vaccine is used in children and a combined tetanus-diptheria ‘Td’ vaccine containing a smaller amount of diphtheria toxoid is recommended for adults instead of tetanus toxoid alone as it will increase population immunity to diphtheria. In the UK primary immunization courses use a combined DTaP/IPV/Hib (diphtheria, tetanus, pertussis, polio, haemophilus influenzae B) vaccine whereas a Td/IPV (tetanus diphtheria polio) vaccine is used in adults.^15^

Neonates are protected from tetanus by passive transfer of maternal antibody across the placenta. Pregnant mothers who have not received full immunization require two dose of tetanus toxoid spaced at least one month apart to generate sufficient antibody for this purpose. A third dose is recommended after delivery to promote long-term immunity. Approximately 80% of maternal antibodies are still present in infants one month after delivery thus protection is maintained until a primary vaccination course is given and is maximal at the most vulnerable period when umbilical infection may occur.^16^

Since 2000, primary vaccination coverage rates in the UK have been >95%. Antibody levels >0.1 IU/l measured by ELISA are taken to be protective against tetanus, although this method of measurement is subject to some limitations and there have been occasional reports of tetanus occurring despite sufficient antibody levels.^16^ In well-resourced settings, the elderly and persons who inject drugs are the main risk groups for tetanus and additional vaccination of these high risk groups has been advocated.^15,17^ In the UK Individuals, born before 1961 may have missed primary vaccination as well as experience declining antibody titres over time. Tetanus vaccination has been given to the Armed Forces since 1938, thus elderly women are particularly at risk. All seven cases occurring in the UK in 2013 received less than the five recommended doses of tetanus toxoid. The use of dirty needles, ‘cutting’ of heroin with contaminated adjuvants and the injection method of ‘popping’ (subcutaneous or intramuscular injection) are suggested to be additional risk factors in persons who inject drugs.^18^

Contaminated injuries should be treated with vaccination with or without antitoxin. Open fractures which are heavily contaminated are at highest risk of tetanus and in addition to thorough cleaning, tetanus antitoxin should be given, as well as vaccination if appropriate.^15,19^ It is recommended that antitoxin should be human immune globulin as this is less likely to result in anaphylactoid reactions than equine-based products. However human antitoxin is more costly and often difficult to obtain, thus in many countries equine antitoxin may be the only option available, although anaphylactoid reactions may be less common than those reported in the earlier studies.^20^ The precise dose of antitoxin is derived from case-series in the 1950s and 1960s and recent guidelines have suggested these could be reduced.^21^ However, in many countries vaccination coverage is low and there is little or no post-exposure treatment for tetanus-prone injuries.

### Neonatal tetanus

Neonatal tetanus in particular has been chosen as the focus of a global prevention project (Box 1). At the end of the 1980s, high rates of disease, with correspondingly high mortality, were seen as unacceptable given the availability of a cheap and effective prevention method (maternal vaccination). The World Health Organization and its partners the United Nations Children's Fund and United Nations Population Fund launched a programme aiming to ‘eliminate’ neonatal tetanus from 57 (later expanded to 59) countries. Neonatal tetanus elimination is defined as less than one case per 1000 live births in every district in a country, thus achieving elimination does not mean complete eradication and countries achieving elimination may still report cases.^22^

Box 1. **Case definitions of neonatal tetanus used in the maternal and neonatal tetanus elimination initiative**

**Table:** 

Suspected case		• Any infant with a history of tetanus-compatible illness during the first month of life who fed and cried normally for the first 2 days of life
	or	• Any neonatal death in a child who could suck and cry normally during the first 48 h of life
Confirmed case		• Normal feeding and crying during the first two days of life
	and	Onset of illness between age 3 and 28 days
	and	Inability to suckle (trismus), followed by stiffness (generalized muscle rigidity) and/or convulsions (muscle spasms)

**Fig. 1:**
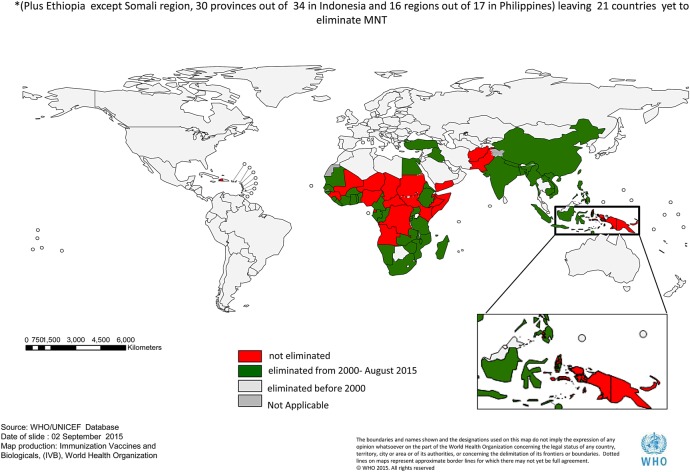
Thirty-eight countries eliminated MNT between 2000 and August 2015.

Considerable progress has been made towards global elimination of neonatal tetanus, with most recent figures reporting 38 of the targeted countries have now eliminated the disease with a reduction in incidence to 58 000 cases a year^23,24^ (Fig. 1). The elimination of maternal tetanus, defined as tetanus occurring during pregnancy or within 6 weeks of any form of termination, was added to the target in 2000. Elimination is assumed to occur alongside neonatal tetanus elimination. There are few data regarding maternal tetanus specifically but a study in 1993 estimated an annual incidence of 15 000–30 000 cases.^25^

The programme involves two main strategies: immunization of women and improving birth hygiene. Initially the programme principally targeted pregnant women, aiming to deliver two doses of tetanus toxoid one month apart during pregnancy (Box 2). Systematic review and meta-analyses have shown that vaccination is the major factor associated with reducing neonatal tetanus mortality with a relative risk estimated of 0.06.^26,27^ A recent study in India indicates that 16% of all neonatal deaths (78 632 cases) is attributable to a lack of maternal immunization with two doses of tetanus toxoid during pregnancy.^28^ Between 1999 and November 2014, 128 million women (out of a targeted 161 million) received two or more doses of tetanus toxoid vaccination.^23^

Box 2. **Strategies employed by the maternal and neonatal tetanus elimination initiative**

Immunization of pregnant women

Supplementary Immunization Activities in selected high risk areas

Promotion of clean deliveries and clean cord care practices

Reliable neonatal tetanus surveillance

More recently, in addition to vaccination in pregnancy, a policy of ‘supplementary immunization activities’ has been employed. These have been used in areas deemed to be at high risk of neonatal tetanus where a conventional approach may not be effective and include activities such as vaccinating all women of child-bearing age. Opportunities for vaccination are extended beyond the conventional antenatal setting for example schools, markets and other community locations. This has allowed significant progress to be made in many areas.^29^ A total of 54 countries have expanded or employed supplementary immunization activities between 1999 and 2013.

Closely linked to vaccination is a policy of enhanced surveillance to detect the incidence of neonatal tetanus more accurately. Neonatal tetanus occurs in remote communities with limited access to and use of healthcare. Studies have indicated that only 2–5% of cases may be reported, making planning and assessment of elimination programmes difficult.^30^ Strengthening of reporting systems has been regarded as a priority in the neonatal tetanus elimination initiative. New approaches that have been used, include the incorporation of neonatal tetanus into flaccid paralysis monitoring in Nepal, allowing weekly reports of neonatal tetanus to be collected from 431 ‘sentinel centres’ throughout Nepal and improving both accuracy and speed compared with conventional methods.^29^ Results fed back in a timely manner allow better planning and implementing of interventions. An investigation following the reporting of three cases of neonatal tetanus in Papua New Guinea led to the design and implementation of a national ‘action plan’ to conduct three rounds of tetanus toxoid vaccination in all women of child-bearing age achieving coverage of 77% in 1.3 million women.^31^

Another component of elimination strategy is improved birth hygiene. The provision of skilled birth attendants and clean delivery facilities has been advocated by the World Health Organization^32^ (Box 3). A systematic review examining the effects of various methods of improved hygiene found that overall the evidence was of low quality but a Delphi consensus process concluded that neonatal tetanus mortality was reduced by 30% with clean practices at home, 38% if birth was in a facility and by 40% with the addition of postnatal care practices.^33^ A recent study from Pakistan reports that both the provision of a skilled birth attendant and a clean birth kit are independently associated with a reduction in neonatal tetanus, and there is a 24% population attributable risk for not using a clean delivery kit.^34^

Box 3. **The six ‘Cleans’ of delivery^32,33^**

Clean hands

Clean perineum

Clean delivery surface

Clean cord cutting

Clean cord tying

Clean cord care

Validation of neonatal tetanus elimination is a staged process. Initially a review of all district-level data is performed, for example number of neonatal tetanus cases and vaccination coverage. In addition any other available data such as results of supplementary immunization activities are examined. From this process elimination in all districts is established and the weakest performing districts are identified. Field visits may be undertaken in areas with limited or uncertain data. Validation surveys are then performed using lot-quality assurance and cluster surveys.^35^ Weakest performing districts are selected as it is expected that other areas are likely to have better outcomes. Finally, once validation is completed a long-term plan is required aiming to ensure ongoing elimination status.

Unfortunately neonatal tetanus occurs in settings remote from health-care facilities and it is likely that surveillance systems under-estimate its true occurrence and similarly that vaccination coverage is over-estimated.^36^

## Areas of controversy

Maintaining tetanus elimination requires continued investment in public health and emergency care. In many countries there is little provision for the booster vaccinations required in childhood and adolescence for long-term immunity.

**Fig. 2:**
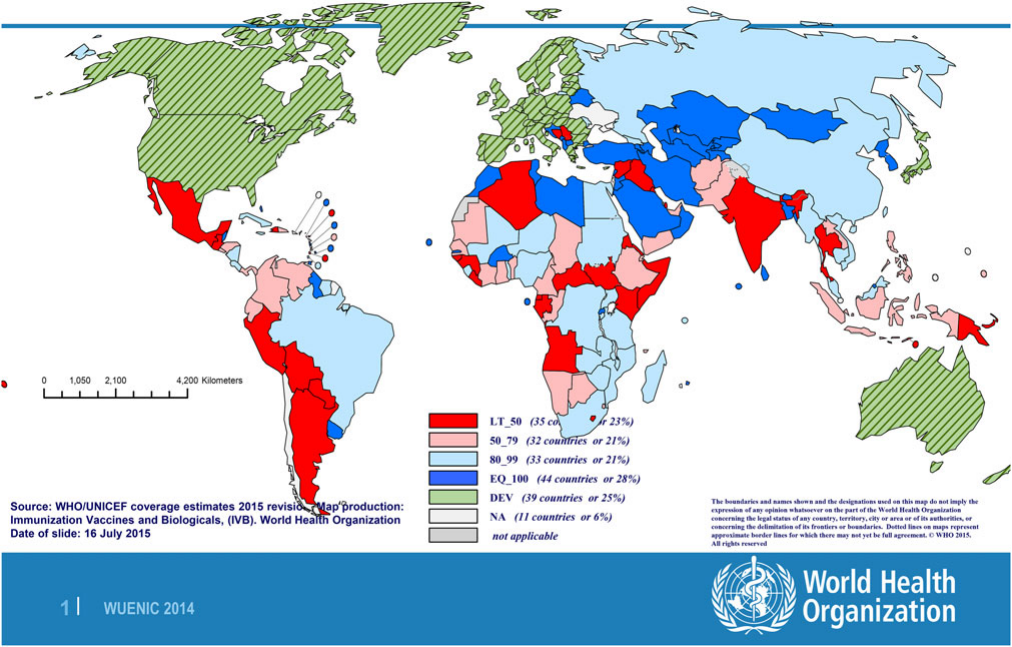
‘Developing’ *countries with % of districts achieving at least 80% DTP3 coverage, 2014. (red = less than 50%, pink = 50–79% light blue 80–99% and dark blue 100% of districts).

Under the World Health Organization's Expanded Programme on Immunization (and World Millennium Development Goal 4) all infants should receive vaccination against tetanus, in combination with diphtheria, pertussis and polio. Currently 96% of children in high income countries receive the recommended primary course, compared with only 72% of those in Africa and 75% in South East Asia.^37^ However, even with full vaccination in infancy only 4–6 years of protection is provided, and boosting is required for long-term immunity^16^ (Fig. 2). A booster dose within 5 years of primary immunization provides protection for up to 20 years and it is likely that a total of 5 doses is sufficient for life-long immunity. In the UK recommendations have removed the requirement for 10-yearly boosters.^38^

However throughout much of the world there is little provision for immunization beyond infancy and very few data to indicate the extent of protection. Data from Tanzania indicate that the Expanded Programme on Immunization schedule results in protective levels of antibody in 97% children aged 1–5 years, but by 6–15 years of age only 54% of children are protected.^16,39^ Neonatal elimination programmes may provide further boosters for women, but it is likely large numbers of men receive no further immunization. Cold-chain equipment and labour accounted for the majority of expenditure in a study examining the cost of routine vaccination in Vietnam, with an overall cost per dose of $0.24.^40^ This compares with the often crippling costs to families and health-care systems of treating established disease in many low and middle income countries.

### Malaria/HIV

Malaria and HIV infection are endemic in many of the countries where tetanus is common. Their effect on response to immunization and transplacental transfer of antibody is still unclear. The response to tetanus vaccine in HIV infected people depends on age and the degree of immunosuppression. A study of 48 adults in the Netherlands showed a reduction in both absolute levels of antibody and reduced response to vaccination compared with HIV negative controls.^41^ Similarly antibody levels were reduced in HIV positive postnatal women in Brazil, even after correction for time since last vaccination. HIV positive subjects responses to booster immunization were reduced compared with HIV negative individuals.^42^

The effect of malaria on vaccination response is unclear. Some studies report no change and some show an attenuated response.^43^ One reason for this may be differences in malarial burden and population immunity to malaria.

Transplacental transfer of protective antibody to neonates may also be affected by malaria and HIV, although past studies have shown inconsistent results. Different methods of malaria detection and failure to control for vaccination history may in partly explain this. In a large recent study in Kenya examining the effect of both HIV and malaria on transfer of antibody, tetanus antibodies were reduced by 52% in HIV positive mothers independent of malaria which reduced transfer by 48%.^44^ In Papua New Guinea, in an area with high falciparum loads, 10% of neonates were found to have sub-protective antibody concentrations despite adequate maternal titres.^45^ In the case of malaria reduced transfer may be due to inflammatory changes within the placenta, thus high parasite loads and chronicity of infection are important factors. In HIV a more general reduction in immune response is postulated.^44^

## Growing points

Efforts to eliminate maternal and neonatal tetanus are ongoing and more countries are expected to reach elimination targets in the near future. Thirty-eight countries have eliminated maternal and neonatal tetanus. India eliminated the disease in 2015 and Union Territories and Indonesia in 30 out of 34 provinces. China was validated as having eliminated the disease in 2012—a task requiring 103 survey teams and visits to over 45 000 households. Methods used to achieve elimination in China were distinct from other approaches as births within health-care facilities and increased uptake of antenatal care were particularly encouraged. This was associated with not only elimination of maternal and neonatal tetanus, but also a significant fall in maternal mortality.^46^

There is a growing awareness about the risk of tetanus following natural disasters, with ‘outbreaks’ of tetanus reported after events such as earthquakes and floods. A cluster of 106 cases were attributed to the Tsunami of 2005 in Aceh, Indonesia and 139 cases following the earthquake in Kashmir in 2005.^47,48^ As tetanus is not transmissible from person to person these apparent outbreaks represent the consequence of a large number of contaminated injuries in a population with low levels of immunity. *C tetani* is more readily isolated from the soil after flooding^49^ raising the possibility that in some cases an increase in environmental *C tetani* in addition to lack of medical care and break-down of public health infrastructure may contribute to outbreaks. The World Health Organization has issued recommendations specifically for humanitarian disasters, advocating both active immunization with tetanus toxoid and passive antitoxin administration combined with wound cleaning, debridement and antibiotics.^50^ In Haiiti, when a marked increase in tetanus cases occurred after the 2010 earthquake, an emergency vaccination programme was initiated targeting children and adults aiming to cover all affected areas.^47^

## Areas timely for developing research

Despite the declining incidence of neonatal tetanus, tetanus is still a significant problem and cases continue to occur in unimmunized individuals throughout the world.^8^ Multi-national action to improve vaccination coverage across all age-groups and introduce effective booster dosing is urgently needed. Currently surveillance systems for non-neonatal tetanus are weak and there are no accurate estimates of the true incidence of tetanus in children and adults.

To improve the effectiveness of vaccination programmes, better understanding about the effect of malaria and HIV on response to vaccination and placental transfer of antibodies is needed to inform about optimal frequency and timing of booster vaccinations.

## Funding

Funding to pay the Open Access publication charges for this article was provided by the Wellcome Trust.

## References

[1] Thwaites CL, Yen LM, Nga NT et al Impact of improved vaccination programme and intensive care facilities on incidence and outcome of tetanus in southern Vietnam, 1993–2002. Trans R Soc Trop Med Hyg 2004;98:671–7. 15363647 10.1016/j.trstmh.2004.01.008

[2] Mull DS, Anderson JW, Mull JD Cow dung, rock salt, and medical innovation in the Hindu Kush of Pakistan: the cultural transformation of neonatal tetanus and iodine deficiency. Soc Sci Med 1990;30:675–91. 2315737 10.1016/0277-9536(88)90253-5

[3] Salinas S, Schiavo G, Kremer EJ A hitchhiker's guide to the nervous system: the complex journey of viruses and toxins. Nat Rev Microbiol 2010;8:645–55. 20706281 10.1038/nrmicro2395

[4] Deinhardt K, Salinas S, Verastegui C et al Rab5 and Rab7 control endocytic sorting along the axonal retrograde transport pathway. Neuron 2006;52:293–305. 17046692 10.1016/j.neuron.2006.08.018

[5] Fishman PS, Carrigan DR Motoneuron uptake from the circulation of the binding fragment of tetanus toxin. Arch Neurol 1988;45:558–61. 3358710 10.1001/archneur.1988.00520290094020

[6] Schiavo G, Benfanati F, Poulain B et al Tetanus and botulinum-B neurotoxins block neurotransmitter release by proteolytic cleavage of synaptobrevin. Nature 1992;359:832–5. 1331807 10.1038/359832a0

[7] Udwadia FE Tetanus. Oxford University Press, 1994.

[8] Verde F, Riboldi G, Zappa C et al An old woman with pressure ulcer, rigidity, and opisthotonus: never forget tetanus! Lancet 2014;384:2266. 25625401 10.1016/S0140-6736(14)61832-8

[9] Alves M, Canoui L, Deforges L et al An unexpected trismus. Lancet 2012;380:536. 22863054 10.1016/S0140-6736(12)60524-8

[10] Public Health England. Tetanus in England and Wales: 2013. https://www.gov.uk/government/publications/tetanus-in-england-and-wales-2013/tetanus-in-england-and-wales-2013 (October 2015, date last accessed).

[11] Sutter RW, Cochi S, Brink E et al Assessment of vital statistics and surveillance data for monitoring tetanus mortality, united states, 1979–1984. Am J Epidemiol 1990;131:132–42. 2403465 10.1093/oxfordjournals.aje.a115466

[12] World Health Organization. Tetanus—2005 Global Figures, Geneva http://www.who.int/immunization_monitoring/diseases/tetanus/en/index.html (October 2015, date last accessed).

[13] Thwaites CL, Beeching NJ, Newton CR Maternal and neonatal tetanus. Lancet 2015;385:362–70. 25149223 10.1016/S0140-6736(14)60236-1 PMC5496662

[14] World Health Assembly Expanded Program on Immunization. Resolution WHA42.32; 1989.

[15] Department of Health Immunisation against infectious disease. Chapter 30 Tetanus. https://www.gov.uk/government/publications/tetanus-the-green-book-chapter-30 (October 2015, date last accessed).

[16] Borrow R, Balmer P, Roper M The immunological basis for immunization series. Module 3: Tetanus update 2006. World Health Organisation 2006.

[17] Beeching NJ, Crowcroft N Tetanus in injecting drug users. BMJ 2005;330:208–9. 15677636 10.1136/bmj.330.7485.208 PMC546055

[18] Hope VD, Palmateer N, Wiessing L et al A decade of spore-forming bacterial infections among European injecting drug users: pronounced regional variation. Am J Public Health 2012;102:122–5. 22095355 10.2105/AJPH.2011.300314 PMC3490555

[19] Department of Violence and Injury Prevention and Disability & Organization, W. H. Prevention and management of wound infection guidance from WHO's department of violence and injury prevention and disability and the department of essential health technologies. 1–3 ww.who.int/hac/techguidance/tools/guidelines_prevention_and_management_wound_infection.pdf (October 2015, date last accessed).

[20] Perey F Progress in tetanus prophylaxis: the advent of human antitoxin. Canadian Med Ass J 1966;94:437–41. PMC1935316 5902706

[21] Health Protection Agency. HPA expert working group interim guidance on the use of tetanus immunoglobulin for the treatment of Tetanus. 1–2 (2013). https://www.gov.uk/government/uploads/system/uploads/attachment_data/file/400084/expert_working_group_interim_guidance_on_the_use_of_tetanus_immunoglobulin_for_the_treatment_of_Tetanus.pdf (October 2015, date last accessed).

[22] Lam PK, Trieu HT, Lubis IN et al Prognosis of neonatal tetanus in the modern management era: an observational study in 107 Vietnamese infants. Int J Infect Dis 2014;33C:7–11. 25499039 10.1016/j.ijid.2014.12.011 PMC4396701

[23] WHO Maternal and Neonatal Tetanus (MNT) elimination. http://www.who.int/immunization_monitoring/diseases/MNTE_initiative/en/index.html (October 2015, date last accessed).

[24] Black RE, Cousens S, Johnson HL et al Global, regional, and national causes of child mortality in 2008: a systematic analysis. Lancet 2010;375:1969–87. 20466419 10.1016/S0140-6736(10)60549-1

[25] Fauveau V, Mamdani M, Steinglass R et al Maternal tetanus: magnitude, epidemiology and potential control measures. Int J Gynaecol Obstet 1993;40:3–12. 8094347 10.1016/0020-7292(93)90765-o

[26] Khan AA, Zahidie A, Rabbani F Interventions to reduce neonatal mortality from neonatal tetanus in low and middle income countries—a systematic review. BMC Public Health 2013;13:322. 23570611 10.1186/1471-2458-13-322 PMC3637612

[27] Blencowe H, Lawn J, Vandelaer J et al Tetanus toxoid immunization to reduce mortality from neonatal tetanus. Int J Epidemiol 2010;39:i102–9. 20348112 10.1093/ije/dyq027 PMC2845866

[28] Singh A, Pallikadavath S, Ogollah R et al Maternal tetanus toxoid vaccination and neonatal mortality in rural north India. PloS One 2012;7:e48891. 23152814 10.1371/journal.pone.0048891 PMC3494717

[29] Vandelaer J, Partridge J, Suvedi BK Process of neonatal tetanus elimination in Nepal. J Public Health 2009;31:561–5. 10.1093/pubmed/fdp039 19443437

[30] Stanfield J, Galazka A Neonatal tetanus in the world today. Bull WHO 1984;62:647–69. 6386211 PMC2536335

[31] Datta SS, Barnabas R, Sitther A et al Three cases of neonatal tetanus in Papua New Guinea lead to the development of national action plan for maternal and neonatal tetanus elimination. WPSAR 2013;4:8–11. 24015370 10.5365/WPSAR.2013.4.1.008 PMC3762966

[32] WHO Making pregnancy safer: the critical role of the skilled attendant. A joint statement by WHO, ICM and FIGO, 2004 http://whqlibdoc.who.int/publications/2004/9241591692.pdf (October 2015, date last accessed).

[33] Blencowe H, Cousens S, Mullany L et al Clean birth and postnatal care practices to reduce neonatal deaths from sepsis and tetanus: a systematic review and Delphi estimation of mortality effect. BMC Public Health 2011;11 Suppl 3:S11. 21501428 10.1186/1471-2458-11-S3-S11 PMC3231884

[34] Raza S, Avan BI Disposable clean delivery kits and prevention of neonatal tetanus in the presence of skilled birth attendants. Int J Gynaecol Obstet 2013;120:148–51. 23261127 10.1016/j.ijgo.2012.07.030

[35] WHO Progress towards global MNT elimination. http://www.who.int/immunization_monitoring/diseases/MNTE_initiative/en/index4.html (October 2015, date last accessed).

[36] Cutts FT, Izurieta HS, Rhoda D Measuring coverage in MNCH: design, implementation, and interpretation challenges associated with tracking vaccination coverage using household surveys. PLoS Med 2013;10:e1001404. 23667334 10.1371/journal.pmed.1001404 PMC3646208

[37] WHO World Health Statistics 2014, 2013 http://apps.who.int/iris/bitstream/10665/81965/1/9789241564588_eng.pdf (October 2015, date last accessed).

[38] Health Protection Agency. Tetanus: information for health professionals. 1–9 (2013). https://www.gov.uk/government/publications/tetanus-advice-for-health-professionals (October 2015, date last accessed).

[39] Aboud S, Matre R, Lyamuya EF et al Levels and avidity of antibodies to tetanus toxoid in children aged 1-15 years in Dar es Salaam and Bagamoyo, Tanzania. Ann Trop Paediatr 2000;20:313–22. 11219170 10.1080/02724936.2000.11748153

[40] Mvundura M, Kien VD, Nga NT et al How much does it cost to get a dose of vaccine to the service delivery location? Empirical evidence from Vietnam's Expanded Program on Immunization. Vaccine 2014; 32:834–8. 24370713 10.1016/j.vaccine.2013.12.029

[41] Kroon F, Van Dissel J, Labadie J et al Antibody response to diphtheria, tetanus, and poliomyelitis vaccines in relation to the number of CD4+ T lymphocytes in adults infected with human immunodeficiency virus. Clin Infect Dis 1995;21:1197–203. 8589143 10.1093/clinids/21.5.1197

[42] Bonetti T, Succi R, Weckx L et al Tetanus and diphtheria antibodies and response to a booster dose in Brazilian HIV-1-infected women. Vaccine 2004;22:3707–12. 15315850 10.1016/j.vaccine.2004.03.023

[43] Brabin BJ, Nagel J, Hagenaars AM et al The influence of malaria and gestation on the immune response to one and two doses of adsorbed tetanus toxoid in pregnancy. Bull WHO 1984;62:919–30. 6335850 PMC2536266

[44] Cumberland P, Shulman CE, Maple PA et al Maternal HIV infection and placental malaria reduce transplacental antibody transfer and tetanus antibody levels in newborns in Kenya. J Infect Dis 2007;196:550–7. 17624840 10.1086/519845

[45] Brair ME, Brabin BJ, Milligan P et al Reduced transfer of tetanus antibodies with placental malaria. Lancet 1994;343:208–9. 7904669 10.1016/s0140-6736(94)90991-1

[46] Liu X, Yan H, Wang D The evaluation of ‘Safe Motherhood’ program on maternal care utilization in rural western China: a difference in difference approach. BMC Public Health 2010;10:566. 20858285 10.1186/1471-2458-10-566 PMC2946300

[47] Afshar M, Raju M, Ansell D et al Annals of internal medicine review narrative review: tetanus—a health threat after natural disasters in developing countries. Ann Int Med 2011;154:329–36. 21357910 10.7326/0003-4819-154-5-201103010-00007

[48] Jeremijenko A, McLaws M, Kosasih H A tsunami related tetanus epidemic in Aceh, Indonesia. Asia Pac J Public Health 2007;19:40–4. 18277527 10.1177/101053950701901S07

[49] Huang SW, Chan JP, Shia WY et al The utilization of a commercial soil nucleic acid extraction kit and PCR for the detection of clostridium tetanus and clostridium chauvoei on farms after flooding in Taiwan. J Vet Med Sci 2013;75:489–95. 23208321 10.1292/jvms.12-0271

[50] World Health Organization. WHO current recommendations for treatment of tetanus during humanitarian emergencies, 2010 http://whqlibdoc.who.int/hq/2010/WHO_HSE_GAR_DCE_2010.2_eng.pdf (October 2015, date last accessed).

